# Active Methodologies in Physical Education: Perception and Opinion of Students on the Pedagogical Model Used by Their Teachers

**DOI:** 10.3390/ijerph18041438

**Published:** 2021-02-03

**Authors:** Emilio Crisol Moya, María Jesús Caurcel Cara

**Affiliations:** 1 Department of Didactics and School Organization, University of Granada, 18071 Granada, Spain; ecrisol@ugr.es; 2 Department of Developmental and Educational Psychology, Faculty of Education, University of Granada, 18071 Granada, Spain

**Keywords:** active methodologies, perception, opinion, physical education, university students

## Abstract

The teaching of physical education today still incorporates innovative methodologies in order to create quality physical education. This article sets out to describe which pedagogical model is used in the initial training of physical education teachers at the University of Granada, from the perspective of the students. The study adopted an exploratory, descriptive and comparative research design, applying a survey to a sample of 303 physical education students. The students perceive that their teachers make use of different organising modalities, methodological strategies and assessment systems that favour the use of active methodologies. The structural equations model for analysing predictive relations between the three methodological components (organising modalities, methodological approaches and evaluation systems) was fitted correctly, obtaining positive relations between the three components. The model also showed positive and negative influences in the opinion of the students in the planning of the teaching–learning methodologies and some of the methodological components. The results indicate that the perception and opinion of the physical education students take on a special role in the development of student-centred methodologies.

## 1. Introduction

Education today has inherited a tradition characterised by a one-size-fits-all methodology [1] aimed at an education focused on content, materials, pacing and method [2].

However, there are other forms of education that seek to reinvent education, such as neuro-education. This is a new vision of teaching that provides educational strategies and technologies that are based on how the brain functions. This educational discipline brings together knowledge on neuroscience, psychology and education with the aim of optimising the teaching–learning process [3] through the creation of innovative teaching methodologies, formed from data provided by neuro-education [4]. 

Through this premise, students are not limited to passively receiving information; rather, they handle it, participating actively in its creation [5]. This idea has led to the promulgation of the concept of active methodology, which is a new form of transmitting and creating knowledge that is shared and developed by the students themselves, under guidance from the teacher for an optimal achievement of objectives [6], and the consolidation of content [7,8,9]. 

In the initial training of physical education teachers, as well as the specific content of the discipline, there is a need to deliberately tackle a range of methodological aspects that are transferable to the classroom and that will develop their professional activity [10]. These are related to teaching plan designs, organising groups, time management and applying assessments. All these aspects are clearly involved in the development of new pedagogical models [11], which are envisaged as facilitating and encouraging critical thought [12], engaging the students with problems from the real world that they will go on to play a part in. Based on an interdependent relationship between teaching, learning, content and context, active methodologies are thus framed within pedagogical models [13]. This approach is supported by the UN, as part of the framework of aims from the 2030 Agenda for Sustainable Development, which propounds an educational model that endeavours to attain the development of student competence through a learning-centred model by way of active methodologies. Conveying a model correctly is as important as learning it. In this way, there is some bridging of the gap that tends to exist between the theoretical evidence in the training guidelines of physical activity and its subsequent use in the actual classroom [14]. Different methodological concepts have emerged along these lines, which take up this type of approach and discourse in the area of physical education, producing proposals that aim to be an alternative to traditional ways of understanding and practising physical education [15]. Therefore, in agreement with the current guidelines from international and national reports, which set out the policies for giving quality physical education, the use of more open methodologies is advocated—open methodologies that involve the active participation of students in their learning, based on context and competences. This means that more traditional models—understood as those focused on teaching and performance—are left behind [5].

In the literature, quite a few publications have over time recommended using the learning-centred model in different areas of knowledge [16,17,18,19,20,21,22,23,24,25,26,27,28], while other studies have analysed its use in practice [29,30,31,32,33,34,35,36,37,38,39,40,41,42,43,44], with many focusing on physical education [5,45,46,47,48,49,50,51,52,53,54,55,56,57]. These studies make it clear that a methodological redevelopment is underway that involves the use of new forms of organisation (modalities of organisation), teaching methodologies (methodological approaches), and assessment processes (assessment systems) [58,59,60,61]. These align with the new professional profiles and a new way of understanding learning that is crucial for the transition from a teaching-based methodology to one based on encouraging active learning [62,63,64].

Of the many broad definitions of active learning, all of them involve more than just passive listening [56,58,63,65,66,67,68,69]. Active learning is a broad term and, in common use, is “generally defined as any instructional method that engages students in the learning process” [70]. 

This new focus of the teaching practice has brought about an increase in motivation [35,36,37,38,39,40,41,42,43,44,45,46,47,48,49,50,51,52,53,54,55,56,57,58,59,60,61,62,63,64,65,66,67,68,69,70,71] and an improvement in student attitude [72], due to the new opportunities and media of learning that they have at their disposal [73], bringing modern education closer to the peculiarities of digital society [74]. This has made it possible not only to make use of new ways of conveying content but also the appearance of new spaces and times for the instructive process [7], which is known as ubiquity [75]. 

The use of active methods in university classrooms is effective as long as the teacher takes student participation into account in the organisation and design of the teaching–learning methodologies, as well as in the assessment methods [64,76,77,78,79]. 

The theory behind the use of active methodologies is based on a constructivist view of learning [79,80,81]. Being constructive means guaranteeing that all the components of the teaching–learning process are developed unanimously, so that both the methodological approaches (teaching methods) and the assessment systems (evaluation procedures) are designed to achieve the desired learning competences and results [63,64].

The problem with this methodology, which encourages active learning, is that it is often poorly applied or not applied at all, with the result that the active methodologies are only actually present in theory. It is not enough that the use of active methodologies confers a very meaningful role to the students, who construct their knowledge from certain guidelines, activities or scenarios designed by the teacher. Rather, through these activities, the teacher should encourage the students [64] to: be responsible for their own learning, and to develop skills of searching, selection, analysis and evaluation of information; to participate in activities that enable them to interchange experiences and opinions with their peers; to commit to processes of reflection about what to do, how to do it and what results to obtain, proposing specific actions for improvement; to interact with their environment in order to partake in it socially and professionally through activities such as projects, case studies and problem solving; and to develop autonomy, critical thinking, attitudes for collaboration, professional skills and the capacity for self-assessment. 

These key issues help to determine how to organise the students’ learning, how to assess them and how both the teacher and the students should act [82,83]. Given that these issues represent the three fundamental components of these methodologies, they formed the main focus of the present study. First is the organisational component, that is, the scenario or scenarios in which the teaching–learning processes are to be developed. In this study, these are determined as modes of organisation following classification [82,83]: theoretical classes; seminars/workshops; practice classes; tutorials; external practice/placements; and individual, independent and team work and study. The second comprises the procedural–technical component, formulated as methodological approaches, structured with [82,83]: participatory lectures, oral presentations of student projects, seminars, case studies, problem-based learning, portfolios, independent work, cooperative work, project-based learning, learning contracts and conceptual maps. The last component is evaluative, defined as systems of assessment [82,83]: objective tests, long-answer tests, oral exams, assignments and projects, reports/notes on practice class sessions, tests on the performance of real tasks, self-assessment systems, observation techniques, portfolios and conceptual maps. For a detailed description of the modalities, methodological approaches and assessment systems, please see the study carried out by Crisol [62]. 

It is not easy to move from an approach based on teaching to one based on learning [63,64,84]. This change requires organisational changes, new infrastructures and teams, cooperative work by teachers, and an integrated study plan design [63,85]. All these changes need the motivation and commitment of teachers and students, as well as training programmes for the teachers [85,86] since they continue to organise and plan around giving lectures. 

This article aims to shed light on what pedagogical model is being used in the initial training of physical education teachers at the University of Granada from the perspective of the students. The objectives of the study are: (a) to learn the students’ opinion on the use of active methodologies; (b) to describe the students’ perception (frequency of use) and opinion (suitability of use) on the modes of organisation, methodological approaches and assessment systems that define the teaching–learning process; (c) to determine differences according to the sociodemographic, academic and relational variables of the participants: age, sex, gender, degree studied, level of satisfaction with the training received, meaning and utility of the new active methodologies, use of active methodologies by the teachers and the opinion of the students on the approach of the teaching–learning methodologies; and (d) to analyse correlations between the variables studied. 

Lastly, we aim to set up a structural equation model (SEM) in order to estimate the possible effects or relations between the different constructs involved in the study, for the purpose of: (a) analysing the existing relations between the three fundamental components of the organisation of the students’ learning: organizational and methodological approaches and assessment systems, as well as with their satisfaction; and (b) analysing the effect that including the students’ opinion has on the approach of the teaching–learning methodologies (modes of organisation, methodological approaches and assessment systems). 

## 2. Materials and Methods

### 2.1. Participants

The population under study was made up of 3068 students, enrolled in the 2017–2018 academic year at the University of Granada (UGR) (UGR Statistics, 2018) in the degrees of Sport and Physical Activity Science (CAFD) (N = 921), of whom 232 were women and 689 were men, and Primary Education (CE) (N = 2147), with 1277 women and 870 men, of whom 170 specialised in physical education (CEPEF) (Comprehensive Student Management System, SIGA, 2017–2018). We used simple random probability sampling [87] to select the participants, producing a total sample of 303 students: 145 from CAFD and 158 from CEPEF. The sociodemographic, academic and relational data of the sample are presented in Table 1: student knowledge, use, satisfaction and opinion on the active methodologies. 

Most of the participants knew the meaning and utility of active methodologies (CAFD N = 80; CEPEF N = 85), stating that their teachers used them (CAFD N = 128; CEPEF N = 117) and that their opinion was included in planning the teaching–learning methodologies (CAFD N = 69; CEPEF N = 66). Furthermore, most students stated that they were quite or very satisfied with the training received (CAFD N = 126; CEPEF N = 101). 

### 2.2. Design of the Study and Instruments

This was a quantitative study with a cross-sectional and descriptive approach. Using an exploratory, descriptive and comparative research model, it explored the opinion of CAFD and CEPEF university students on the use of active methodologies. It described the perception of these two groups of the different modes of organisation, methodological approaches and assessment systems.

The study was developed within the framework of an analytic–synthetic method, with its starting point being the use of the questionnaire as a research instrument to approximate to reality in an objective and generalisable way. 

We used the questionnaire “Opinion y Perception de los estudiantes sobre el uso de metodologías activas en la Universidad de Granada (OPEUMAUGR)” (“Opinion and Perception of students on the use of active methodologies in the University of Granada”) [62,88] comprising 92 items and divided into two parts. The first part, which evaluated “Opinion on active methodologies”, is made up of 43 items in 4 factors: methodological redevelopment (Factor I) (12 items), which analysed the actions that determined the methodological change in the university; use of active methodologies (Factor II) (9 items), which evaluated how the active methodologies were put into practice; context in the university (Factor III) (4 items), which focused on the aspects that characterised the habitual teaching–learning process; and context in the university classrooms (Factor IV) (8 items), which referred to aspects characterising the teaching in the university classrooms. It uses a four-point Likert-type scale: 1, “Completely disagree”, 2 “Disagree”, 3 “Agree” and 4 “Completely agree”. The second part of the questionnaire, which analysed “Perception and opinion on the teaching–learning process”, had 60 items divided into three dimensions: modes of organisation (14 items), methodological approaches (22 items) and sssessment systems (22 items). For “Frequency of use (perception)”, the following response scale was used: 1 “Not at all”, 2 “Little”, 3 “Quite” and 4 “A lot”. For “Suitability of use (opinion)”, the response options were: 1 “Completely unsuitable”, 2 “Not very suitable”, 3 “Suitable” and 4 “Very suitable”. 

The instrument presented adequate psychometric properties, with values in the normed fit index (NFI) of 0.894, Tucker–Lewis index (TLI ) of 0.810, comparative fit index (CFI) of 0.848 and root mean square of residuals (RMSR) of 0.066, which indicate an adequate fit of the instrument and an acceptable model [89,90], and a Cronbach’s alpha coefficient of 0.920, with a reliability level of 95% (*p* ≤ 0.05) for the global scale, being 0.899 for the participants of this study [62,91]. 

### 2.3. Procedure

To ensure fidelity and responsible research, the study followed the ethical considerations established by the ethics committee of the research team’s university (nº 192/CEIH/2020). The study was made up of the following phases. First, the sample was determined and the selection made. Then a meeting was held with the teachers of the degree courses to gain access to the sample. The teachers gave permission for the study to go ahead, which enabled us both to select the sample and to create and complete the instrument with the consequent informed consent of the participants. 

In the second phase, which was accessing different classrooms, the researchers informed the students about the study objectives and the voluntary and anonymous nature of participation. They explained the procedure to access the questionnaire online on Google Forms, and they handed out in writing the access link and numerical password valid for a single use. Subsequently, they read out the instructions—also included on the questionnaire itself—for completing the questionnaire and potential doubts were resolved. The students who wished to fill out the questionnaire had one week to do so. 

### 2.4. Data Analysis

For the data analysis, the IBM SPSS version 26 (IBM, Madrid, Spain) and IBM SPSS Amos version 24 (IBM, Madrid, Spain) statistical packages were used. First, on the study objectives, we carried out descriptive (mean and standard deviation) and frequency analyses to characterise the sample and the opinion of the students from the area of physical education (CAFD and CEPEF) about active methodologies. Second, we applied normality and homoscedasticity tests to the sample, which allowed us to proceed with parametric statistics. Third, we calculated the Pearson correlation to find out the existing relation between the different factors that measure the opinion on active methodologies. Then, in order to study the comparisons between groups, we did a *t*-test for independent samples and a univariate ANOVA with the Bonferroni multiple comparisons test, in order to define between which groupings significant differences were observed according to the variables studied. Lastly, we used a structural equation model to estimate the effects or relations between the different constructs involved in the study, employing the IBM SPSS Amos version 24 program.

## 3. Results 

### 3.1. Opinion on Active Methodologies 

The descriptive results of the students’ opinions of active methodologies revealed that the highest degree of agreement occurred in the use of active methodologies (Factor II) (M = 3.60, SD = 0.656) and context in the university (Factor III) (M = 2.92, SD = 0.491). In contrast, they showed the least degree of agreement in Factor IV (M = 2.72, SD = 0.354), related to the context in the university classrooms and Factor I (M = 2.68, SD = 0.342), concerning methodological renewal. 

The correlation analysis showed that there was a positive and significant association (*p* < 0.01) between the responses on the use of active methodologies (Factor II) and methodological redevelopment (Factor I) (r = 0.297). A positive and significant (*p* < 0.01) relation was found between the responses on the context in the university (Factor III) and methodological renewal (Factor I) (r = 0.485) and the use of active methodologies (Factor II) (r = 0.176). Similarly, a direct and significant (*p* < 0.01) relation was also found between the responses concerning the context in the university classrooms (Factor IV) and methodological renewal (Factor I) (r = 0.497), the responses obtained on the use of active methodologies (Factor II) (r = 0.229) and the context in the university (Factor III) (r = 0.807).

No differences were found in the students’ opinions as a function of age, and only marginally significant differences in Factor III according to sex and gender: t(300) = −1.964; *p* = 0.05. In this case, the women—who identified with the female gender—showed a greater degree of agreement with the questionnaire statements referring to the context in the university (M = 3.03, SD = 0.526), compared to the men (M = 2.91, SD = 0.541). 

Statistically significant differences were observed in Factor II as a function of which degree the students were studying (t(231.715) = 1.989; *p* < 0.05) with the CAFD students (M = 2.68, SD = 0.404), showing a higher degree of agreement with the questionnaire statements referring to the use of active methodologies, in comparison with the CEPEF students (M = 2.53, SD = 0.818).

Regarding whether the students knew the meaning and utility of active methodologies, statistically significant differences were found in the opinions referring to Factor II (t(298) = 2.437; *p* < 0.05) and in total (t(296) = 2.321; *p* < 0.05). The students that knew the meaning and utility of the active methodologies showed a greater degree of agreement (M_Yes_ = 3.04, SD = 0.471 vs. M_No_ = 2.73, SD = 0.335) with the questionnaire statements referring to the context in the university, and also showed, in general, a greater degree of agreement with the statements on active methodologies (M_Yes_ = 2.79, SD = 0.324 vs. M_No_ = 2.70, SD = 0.319). With respect to the use of active methodologies by their teachers, statistically significant differences were obtained in Factors I (t(78.138) = 2.740; *p* < 0.01), II (t(103.462) = 2.393; *p* < 0.05) and III (t(71.645) = 2.470; *p* < 0.05) and in total (t(74.643) = 2.775; *p* < 0.01). Once again, the students that showed that their teachers used active methodologies expressed a higher degree of agreement with the questionnaire statements on methodological renewal (M_Yes_ = 2.71, SD = 0.327 vs. M_No_ = 2.56, SD = 0.380), use of active methodologies (M_Yes_ = 2.64, SD = 0.678 vs. M_No_ = 2.44, SD = 0.529) and context in the university (M_Yes_ = 2.96, SD = 0.446 vs. M_No_ = 2.75, SD = 0.622), as well as in general on the active methodologies (M_Yes_ = 2.78, SD = 0.291 vs. M_No_ = 2.64, SD = 0.373).

According to the students’ level of satisfaction with the training received, statistically significant differences were only obtained in Factor I (F(24, 278) = 8.517; *p* < 0.001) and in total (F(52, 247) = 4.362; *p* < 0.01). The students who were quite (M = 2.66, SD = 0.323) or very satisfied (M = 2.87, SD = 0.384) with the training received showed a higher degree of agreement with the questionnaire statements that referred to the methodological redevelopment, compared to those students who were a little (M = 2.55, SD = 0.367) or not very satisfied (M = 2.54, SD = 0.000). Similarly, the students who were very satisfied (M = 2.88, SD = 0.341) with the training received showed a higher degree of agreement with the questionnaire statements on active methodologies, in comparison with the students who were a little satisfied (M = 2.66, SD = 0.384).

Taking into consideration the opinion of the students in the planning of the teaching–learning methodologies), statistically significant differences were seen in Factors I (t(286.454) = 4.637; *p* < 0.001), III (t(297.976) = 1.725; *p* < 0.05) and in total (t(294.987) = 3.325; *p* < 0.01). The students who affirmed that their teachers took their opinion into account when setting out the teaching–learning methodologies were more in agreement with the statements referring to methodological redevelopment (M_Yes_ = 2.78, SD = 0.313 vs. M_No_ = 2.60, SD = 0.330) and context in the university (M_Yes_ = 2.98, SD = 0.436 vs. M_No_ = 2.87, SD = 0.529), as well as in general to the active methodologies (M_Yes_ = 2.82, SD = 0.278 vs. M_No_ = 2.70, SD = 0.332). 

### 3.2. Perception and Opinion on the Teaching–Learning Process 

The descriptive results of the students’ perception (frequent use) and opinion (suitable use) regarding the three components of the teaching–learning process—organisational and methodological approaches, and assessment systems—are presented in Table 2. According to the students’ perception, the organisational modalities frequently used by the teachers were theoretical classes (3.20) and group study and work (3.00), while the least used was individual self-directed study and work (1.81). However, according to the students, the ideal modalities were the tutorials (3.45), individual, self-directed study and work (3.24) and external practice (3.13).

The methodological strategies frequently used by their teachers were self-directed work (3.24) and cooperative work (3.05), both being considered ideal by the students (3.14 and 3.42, respectively), along with project-oriented work (3.11). With respect to the assessment methods, the students indicated assignments and projects (3.18) and real and/or mock task-performance tests (3.04), with the one scoring the highest as the most suitable being assignments and projects (3.08). 

As a function of degree studied, statistically significant differences were found in the perception and opinion of the organisational modalities (Table 3). The CEPEF students revealed that they had a more frequent use of seminars/workshops, while the CAFD students used tutorials the most. According to their opinion, differences were observed in the seminars/workshops, practice classes, tutorials and individual, self-directed study and work. The CEPEF students considered all these modalities except tutorials to be more ideal.

The degree studied also proved to be discriminating in the perception and opinion on methodological strategies (Table 4). The CAFD students perceived a greater use of presentations, cooperative work, project-based learning and learning contracts, whereas the CEPEF students indicated that participatory master lectures, seminars and portfolios were used more often. According to the CEPEF students’ opinion, participatory master lectures, seminars, cooperative work and project-oriented learning were more suitable, while the CAFD students responded that presentations and learning contracts were more appropriate.

Lastly, with respect to the perception and opinion on the assessment systems, statistically significant differences were also observed according to the degree studied (Table 5). The CAFD students perceived greater use of oral tests, assignments and projects, task-performance tests, self-assessment systems and observation techniques, whereas the CEPEF students only indicated using short-answer tests frequently. Regarding their opinions, the CAFD students considered oral tests, assignments and projects, real and/or mock task-performance tests, observation techniques and conceptual maps to be the most suitable. For their part, the CEPEF students opined that portfolios were the most suitable. 

In respect to the structural equation model (SEM), used to estimate the effects or relations between the different constructs involved in the study, a path analysis was carried out, with the following observable variables: Opinion (OP); Satisfaction (STF); Perception Modalities (PMO); Opinion Modalities (OMO); Perception Methodological Approaches (PMA); Opinion Methodological Approaches (OMA); Perception Assessment Systems (PAS); and Opinion Assessment Systems (OAS).

The suitability tests of the data for preparing the SEM confirmed their univariate normality [90,91]. The model evaluation results indicated a good overall fit in all the indices. A chi-squared test gave a significant associated *p*-value (χ2 = 801,248; df =14; *p* = 0.001). The comparative fit index (CFI) presented a value of 0.945. The NFI value (0.917) was higher than the recommended value of 0.90. The incremental fit index (IFI) value also obtained acceptable values (0.920). Lastly, the root mean square error of approximation (RMSEA) value of 0.053 fit the established parameters [91,92,93,94]. 

The estimations established in the trajectory analysis had significant values, with a positive and significant bidirectional influence (Table 6): between PMA and PAS; between PMA and OMA; between PAS and OAS; between OMA and OAS; between PMO and PMA; between PMO and PAS; between OMA and OMO; and between OAS and OMO. There was also a positive and significant influence between PMA and OP and a negative and significant influence between PAS and OP. 

**Figure 1:**
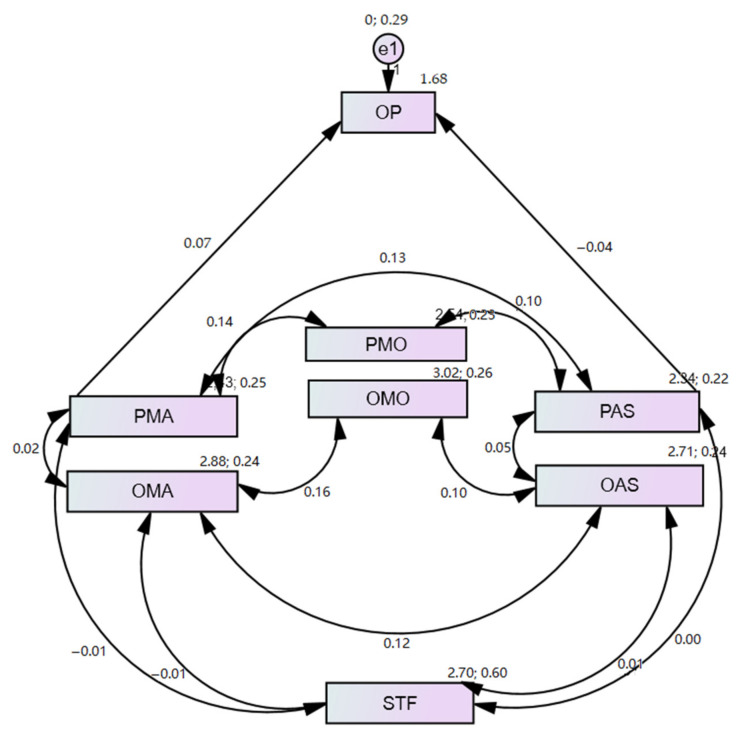
Estimates of the structural equation model. Note: OP = Opinion; STF = Satisfaction; PMO = Perception Modalities; OMO = Opinion Modalities; PMA = Perception Methodological Approaches; OMA = Opinion Methodological Approaches; PAS = Perception Assessment Systems; OAS = Opinion Assessment Systems.

The path analysis graphically brought together the associations between the study variables (Figure 1). The main constructs were PMO, OMO, PMA, OMA, PAS and OAS, with relations of three fundamental components on the organisation of the students’ learning being established between the three types of perceptions and opinions (organisational modalities, methodological approaches and assessment systems), and in turn between the perception and opinion of each component of students’ learning organisation. The opinion (OP) (when the teachers include the opinion of the students in the planning of the teaching–learning methodologies) was positively influenced by the PMA and negatively by the PAS. A relation was also revealed between the STF and the OMA and OAS. These results indicate that the perception the students held of the methodological approaches that their teachers used frequently was correlated with the opinion (suitable use) they held on these methodological approaches. In other words, the perception that the students stated having could arise from their opinion or vice versa.

Moreover, the students’ perception of the frequent use of methodological strategies by their teachers was affected when the teachers took their opinion into account when planning the teaching and learning process. In contrast, taking the students’ opinion into account when determining the planning of the teaching–learning process did not affect their perception of the assessment systems or methods. These results show that the students’ opinion on the planning of the teaching–learning processes only had an influence on the methodological approaches and not on the assessment methods that the teachers would put into practice.

The SEM also showed that when the students’ opinion was taken into account in the planning of the teaching–learning process, their satisfaction regarding the teaching increased.

## 4. Discussion

The implementation of active methodologies offers many possibilities for achieving quality physical education. There is often a “gap” between the theoretical evidence given in the physical education training guidelines and its subsequent use in reality, which leads to a failure to achieve a conscious and rational application of the different physical exercises. Some habits and beliefs about physical activity are distorted and incorrect. This could bring about not only a reduction in participation in physical activity programmes but also cause injuries due to excessive use and poor habits among those who undertake physical activity [95,96]. In this study, we have looked at how the students consider active methodologies, with the aim of raising the awareness of the scientific community of the potential of the combination of active methodologies (organisational modalities, methodological approaches and assessment systems) in the teaching and learning process in the field of physical education. 

The study shows the perception and opinion of CAFD and CEPEF students on the use of active methodologies. These results can help the university community to improve their teaching practice, since it contributes to knowledge on the perception (frequency of use) and opinion (suitability of use) that students have of the teaching and learning processes. Moreover, through this study, we have tested a structural equation model for analysing the predictive relations between the three fundamental components (organisational modalities, methodological approaches and assessment systems) and with the satisfaction and opinion variable of the students in the approach and planning of the teaching–learning methodologies, with the intention of determining how to organise and assess the students’ learning, and the performance both of the teacher and the students. 

Regarding the opinion on active methodologies, in general, the students, and in particular the CAFD students, are in agreement with all the aspects referring to the use of active methodologies. Furthermore, the highest standard deviation value was concentrated in the opinions shown, which had greatest consolidation in the response option. In this case, they agreed that: the active methodologies focused more on the learning of the student than the teaching of the teacher; they enabled the student to confront real problems similar to those that exist in professional teaching; and they encouraged student participation in the classroom as well as content interdisciplinarity. It was also suggested that the use of active methodologies heightened the acquisition of self-directed learning; or that the use of these methodologies fostered group work and learning among the students. 

These results are in line with those from other studies that show that students in general have a positive attitude toward active learning, particularly when the students are informed about the use of active methods [54,55,56,57,58,88]. Ventosa [97] obtains similar results, highlighting that the use of active methods promotes analysis and reflection in the students, contributing to their playing an active role in knowledge acquisition. Perhaps the main difficulty in applying these methods is the large number of students per class, which hinders the undertaking of active methodologies [5,45,55,58,98,99]. 

The results revealed a relation between methodological redevelopment regarding the use of active methodologies and suitability for putting them into practice both at the university and classroom level. This leads to us to determine that, from the students’ point of view, in the area of physical education, different methodological adaptations are being carried out in the teaching and learning process as a response to the demands and needs of implementing active methodologies. 

However, it cannot be said that there was unanimous perception and opinion in all the respondents, as statistically significant differences were found in all the variables studied, except age and sex. This indicates that, in the students’ opinions, the following have an effect: whether they know the meaning and utility of employing active methodologies; whether or not their teachers use active methodologies; their level of satisfaction with the training received; and, of course, whether the teachers take into account their opinion when it comes to determining the planning of the teaching–learning methodologies. 

From this perspective, it is worth reflecting on the importance of methodological renewal, both at the university level as a whole and at the classroom level, in favour of the use of active methodologies, since these measures will contribute to offering a more robust and all the more satisfactory training for the students. This is in line with the argument made by Pérez-López et al. [100] that an ever higher percentage of physical education teachers are trying to introduce methodological adaptation to the teaching–learning process in respond to new social needs and demands, as occurs in their considering student opinion when setting out methodologies. Although there has not yet been complete implementation in the classroom, there is progress toward quality physical education, which upholds the use of more open methodologies and entails the active participation of students in their learning—contextualised and competence based—progressively leaving behind the more traditional models [12,19,20,21,52,53,54,55,100].

With respect to the students’ perception (frequent use) of the learning process, they observe that their teachers made use of different organisational modalities, methodological strategies and assessment systems that favour the use of active methodologies. 

In terms of organisation, the students perceive a varied use of the different modalities, mainly with a continued predominance of theoretical classes. Along with these, the CAFD students stated that there was greater use of tutorials and group study and work, while the CEPEF students observed a higher use of seminars.

Concerning methodological strategies, it is noteworthy that though the use of participatory master lectures was still perceived, it was no longer the principal methodological strategy. In this case, both the CEPEF and the CAFD students indicated that their teachers used self-directed work to a greater extent; the latter also added the use of cooperative work. In the perception of assessment systems, both sets of degree students perceived the use of assignments and projects. However, there were discrepancies, since the CAFD students also identified real or mock task-performance tests and observation techniques, while the CEPEF students highlighted a greater use of short-answer tests and assignments and projects. 

Faced with these data, in accordance with the views of some authors [50], we can still discern certain scepticism on the part of the teachers of the subject, who have feelings of fear or insecurity when it comes to confronting the challenge of changing to a more open and diverse teaching–learning model. Perhaps the uncertainty of giving up some of their responsibility in the intervention, and the lack of experience and/or the need for a specific training of the teachers to implement them, partly explain this feeling of rejection. This conclusion may lead some teachers to not apply active methodologies or to apply them in an indecisive way along with a more traditional methodology (mixed methodology) [49,50]. 

It is worth noting that the students’ perception and opinion coincide with respect to different modalities, methodological strategies and assessment systems for learning physical education. Hence, the CAFD students were in agreement when indicating tutorials and group work as organisational modalities; self-directed work and cooperative work as methodological strategies; and assignments and projects as assessment systems [101,102]. The CEPEF students, however, identified a frequent and suitable use of seminars as organisational modalities, self-directed work as a methodological strategy and assignments and projects as an assessment system. These results are similar to those obtained in comparable studies [13,45,48,60,96,97].

Regarding the predictive relations of the variables studied through the SEM, the structural model had a good fit. We were able to determine that the opinion (OMO, OMA and OAS), that is, the suitable use both of organisational modalities, methodological strategies and also assessment systems, could come to depend on the students’ perception (PMO, PMA and PAS) (frequent use) of them. Dependence on the perception of the methodological approaches was also shown, as a function of whether the teachers considered the students’ opinion in planning the teaching–learning methodologies. However, satisfaction was related neither to perception nor to the opinion of the methodological strategies or assessment systems. This was despite the fact that in different studies [98,100,102,103], the application of an active methodology gains greater satisfaction for the subject.

The structural equation model also established that the frequent use of the modes of organisation, methodological approaches and assessment methods indicated by the students determined their evaluation of suitable use. Likewise, their opinion influenced their perception. Furthermore, the SEM estimated that the fact of taking the students’ opinion into account in determining the teaching–learning processes affected their perception of the methodological approaches, but not of the assessment systems. This result is relevant because for an appropriate and complete transition from a methodology based on teaching to one based on fostering active learning, the teachers need to take their students’ opinion into account [62,64,76]. Moreover, taking the students’ opinion into account in the teaching–learning process brings about an increase in student motivation and satisfaction, due to the new opportunities and media of learning that they have at their disposal [72,73].

Nevertheless, it should be noted that this study has certain limitations. One is its cross-sectional nature, since it only represents that sample in that moment. Additionally, the study was carried out only with students specialising in physical education and through simple random probability sampling. A longitudinal study with the total population could lead to obtaining data that could be assumed to be representative. Furthermore, the present structural equation model has some limitations, including the fact of having analysed the effect and relation of two of the studied variables (students’ opinion of the teaching–learning methodology and satisfaction) without including the other variables. This means not offering the full verisimilitude of the SEM predictive power in comparing with the other variables. In future studies, it would be interesting to complete this analysis by including variables such as the degree studied, academic year, age and sex (gender), as well as carrying out a contrast between the students’ perceptions and the pedagogical models implemented by the teachers.

## 5. Conclusions

Modern education seeks to incorporate new teaching models, enabling a redevelopment of teaching methodologies that transform the learning process into meaningful experiences for the students, especially encouraging aspects such as reflection and active participation in classes [103]. Therefore, what is desired is an approach that enables the students to develop their skills; an approach in which they are the real protagonists, in place of the explicit teaching of teachers [104]. The teaching of physical education should introduce a process of constant change in the students’ teaching practice, with continual transformations [105].

The content of these results show that the teachers are progressing toward a learning-based model, according to the perception and opinion of the students. Equally, given the lack of training that the teachers perceive, we should consider whether the implementation of these innovative proposals in the classroom is only due to a trend that is being carried out without taking into account the principles at the basis of physical education, or the impact on student learning [105]. As Zapatero-Ayuso [106] states, physical education “has educational models at its disposal that aim to attain greater student motivation, participation, autonomy and responsibility through the development of the subject’s content”. Some of these approaches are the Sport Education Model [107], the “Ludotechnical” Model of Sport Initiation, the Teaching Games for Understanding Model and Hellison’s Model of Teaching Personal and Social Responsibility [108]. Therefore, physical education professionals should examine these models, designed for the subject, in order to guarantee the potential benefits of a student-centred intervention.

In light of the results, it is clear that physical education students require a break from traditional methodologies and demand new ways that will give them greater prominence. Moreover, there is no doubt that one of the aims of physical education today lies in responding to the needs of twenty-first century society [109]; the use of active methods is one of the ways of achieving this.

Finally, it should be stated that although a student-based methodology can attain certain benefits [110,111], there is no one better or worse style, as they are all dependent on adopting a critical perspective according to the teaching context [112,113].

## Figures and Tables

**Table 1:** Sociodemographic, academic and relational data of the 303 students evaluated.

Variables		CAFD (N = 145)	CEPEF (N = 158)
Age	18–22 years	129 (89%)	106 (67.1%)
	23–27 years	11 (7.6%)	35 (22.2%)
	Over 28 years	5 (3.4%)	17 (10.8%)
Sex	Female	41 (28.3%)	124 (78.5%)
	Male	104 (71.7%)	34 (21.5%)
	Other	0 (0%)	0 (0%)
Gender	Woman	41 (28.3%)	124 (78.5%)
	Man	104 (71.7%)	34 (21.5%)
	Other	0 (0%)	0 (0%)
Academic year	1st	30 (20.7%)	35 (22.2%)
	2nd	42 (29.0%)	41 (25.9%)
	3rd	45 (31.0%)	49 (31.0%)
	4th	28 (19.3%)	33 (20.9%)
Work	Yes	28 (19.3%)	33 (20.9%)
	No	116 (80.7%)	125 (79.1%)
Meaning and use of the active methodologies	Yes	80 (55.2%)	85 (55.8%)
	No	65 (44.8%)	72 (44.2%)
Use of active methodologies by their teachers	Yes	128 (88.3%)	117 (74.1%)
	No	17 (11.7%)	41 (25.9%)
	None	0 (0%)	2 (1.3%)
Level of satisfaction with training received	Not very	19 (13.1%)	55 (34.8%)
	Quite	97 (66.9%)	85 (53.8%)
	Very	29 (20%)	16 (10.1%)
Opinion of methodological approach	Yes	69 (47.6%)	66 (41.8%)
	No	76 (52.4%)	92 (58.2%)

Note: CAFD = Degree of Sport and Physical Activity Science; CEPEF = Degree of Primary Education, specialising in physical education.

**Table 2:** The students’ perception (frequent use) and opinion (suitable use) on organisational modalities, methodological approaches and assessment methods (N = 303).

Organisational Modalities	Perception		Opinion	
	M	SD	M	SD
Theoretical classes	3.20	0.776	2.90	0.832
Seminars/Workshops	2.28	0.937	2.85	0.916
Practice classes	2.25	1.018	2.95	0.926
Tutorials	2.95	0.935	3.45	0.789
External practice	2.78	0.984	3.13	0.881
Individual, self-directed study and work	1.81	0.991	3.24	0.903
Group study and work	3.00	0.916	3.05	0.807
**Methodological Approaches**				
Participatory master lecture	2.82	0.864	2.93	0.872
Presentation by students	2.64	0.960	2.91	0.934
Seminars	2.22	0.910	2.82	0.888
Case studies	2.10	0.907	2.89	0.911
Problem-based learning	2.24	0.941	3.13	0.800
Portfolio	2.28	1.013	2.66	1.532
Self-directed work	3.24	0.756	3.14	0.779
Cooperative work	3.05	0.873	3.42	1.379
Project-based learning	2.26	0.923	3.11	0.835
Learning contract	2.24	1.017	2.90	0.921
Conceptual maps	2.74	1.061	2.91	0.956
**Assessment Methods**				
Objective tests	2.76	0.861	2.66	1.013
Short-answer tests	2.91	0.810	2.70	0.949
Long-answer, development tests	2.56	0.915	2.59	1.079
Oral tests	2.69	0.952	2.14	1.009
Assignments and projects	3.18	0.825	3.08	0.857
Practice reports/notes	2.87	0.863	2.55	0.957
Real or mock task-performance tests	3.04	0.844	2.53	1.012
Self-assessment systems (oral, written, individual, group)	2.85	0.889	2.37	1.008
Observation techniques	2.89	0.856	2.44	0.991
Portfolio	2.58	0.950	2.22	0.998
Conceptual maps	2.79	0.956	2.59	1.035

Note: M = mean; SD = standard deviation.

**Table 3:** Comparison of the perception and opinion on the organizational modalities as a function of degree studied.

Organisational Modalities	Perception			Opinion		
	CAFD	CEPEF	t	CAFD	CEPEF	t
	M(SD)	M(SD)		M(SD)	M(SD)	
Theoretical classes	3.13 (0.815)	3.27 (0.735)	−1.554	2.95 (0.834)	2.85 (0.831)	1.072
Seminars/Workshops	2.15 (0.996)	2.40 (0.867)	−2.315 *	2.69 (0.995)	3.00 (0.814)	−2.919 **
Practice classes	2.27(1.079)	2.22 (0.962)	0.448	2.82 (0.983)	3.08 (0.856)	−2.414 *
Tutorials	3.22 (0.916)	2.72 (0.889)	4.824 ***	3.55 (0.719)	3.37 (0.840)	1.969
External teaching practice	2.70 (0.975)	2.85 (0.989)	−1.384	3.05 (0.922)	3.21 (0.837)	−1.577
Individual, self-directed study and work	1.86(1.022)	1.77 (0.964)	0.759	3.08 (0.943)	3.39 (0.843)	−3.005 *
Study and work in group	3.08 (0.953)	2.94 (0.879)	1.325	3.13 (0.749)	2.99 (0.852)	1.491

Note: CAFD = Degree of Sport and Physical Activity Science; CEPEF = Degree of Primary Education specialising in physical education; M = mean; SD = standard deviation; t = Student’s *t*-test; * *p* < 0.05; ** *p* < 0.01; *** *p* < 0.001.

**Table 4:** Comparison of the perception and opinion on the methodological approaches as a function of degree studied.

Methodological Approaches	Perception			Opinion		
	CAFD	CEPEF	t	CAFD	CEPEF	t
	M(SD)	M(SD)		M(SD)	M(SD)	
Participatory master lecture	2.67 (0.859)	2.96 (0.847)	−2.923 **	2.84 (0.904)	3.01 (0.837)	−2.923 **
Student presentation	2.85 (0.910)	2.46 (0.968)	3.639 ***	2.95 (0.905)	2.88 (0.960)	3.639 ***
Seminars	2.06 (0.893)	2.36 (0.904)	−2.919 **	2.70 (0.966)	2.93 (0.799)	−2.919 **
Case studies	2.08 (0.949)	2.12 (0.870)	−0.402	2.79 (0.966)	2.97 (0.852)	−0.402
Problem-based learning	2.28 (0.936)	2.20 (0.947)	0.802	3.04 (0.895)	3.21 (0.696)	0.802
Portfolio	2.15 (0.992)	2.39(1.027)	−2.086 *	2.59 (2.001)	2.72 (0.930)	−2.086 *
Self-directed work	3.25 (0.785)	3.23 (0.731)	0.232	3.19 (0.780)	3.09 (0.777)	0.232
Cooperative work	3.22 (0.854)	2.89 (0.864)	3.289 **	3.39 (0.815)	3.44(1.739)	3.289 **
Project-based learning	2.48 (0.930)	2.06 (0.872)	4.004	3.04 (0.890)	3.16 (0.781)	4.004 ***
Learning contract	2.40 (1.101)	2.09 (0.915)	2.563	2.92 (0.942)	2.88 (0.905)	2.563 *
Conceptual maps	2.67 (1.060)	2.80(1.062)	−1.116	2.97 (0.922)	2.85 (0.985)	−1.116

Note: CAFD = Degree of Sport and Physical Activity Science; CEPEF = Degree of Primary Education specialising in physical education; M = mean; SD = standard deviation; t = Student’s *t*-test; * *p* < 0.05; ** *p* < 0.01; *** *p* < 0.001.

**Table 5:** Comparison of the perception and opinion on the assessment systems as a function of degree studied.

Assessment Systems	Perception			Opinion		
	CAFD	CEPEF	t	CAFD	CEPEF	t
	M(SD)	M(SD)		M(SD)	M(SD)	
Objective tests	2.53 (1.035)	2.78 (0.982)	−2.138 *	2.74 (0.967)	2.77 (0.756)	−0.271
Short-answer tests	2.43 (0.968)	2.94 (0.867)	−4.737 ***	2.85 (0.886)	2.97 (0.735)	−1.237
Long-answer, development tests	2.51 (1.021)	2.66 (1.126)	−1.258	2.55 (0.914)	2.56 (0.920)	−0.095
Oral tests	2.42 (1.067)	1.89 (0.886)	4.624 ***	2.90 (0.928)	2.51 (0.938)	3.612 ***
Assignments and projects	3.28 (0.849)	2.91 (0.828)	3.841 ***	3.37 (0.760)	3.02 (0.848)	3.738 ***
Practice reports/notes	2.53 (0.992)	2.56 (0.927)	−0.312	2.91 (0.886)	2.84 (0.844)	0.723
Real and/or mock task-performance tests	2.88 (0.963)	2.22 (0.)953	5.966 ***	3.21 (0.818)	2.89 (0.841)	3.330 **
Self-assessment systems	2.56 (1.012)	2.20 (0.976)	3.138 **	2.83 (0.886)	2.87 (0.894)	−0.423
Observation techniques	2.69 (0.936)	2.22 (0.988)	4.213 ***	3.01 (0.862)	2.78 (0.840)	2.329 *
Portfolio	2.15 (1.010)	2.29 (0.986)	−1.219	2.43 (0.995)	2.70 (0.892)	−2.458 *
Conceptual maps	2.56 (1.047)	2.61 (1.027)	−0.360	2.90 (0.966)	2.68 (0.938)	1.970

Note: CAFD = Degree of Sport and Physical Activity Science; CEPEF = Degree of Primary Education specialising in physical education; M = mean; SD = standard deviation; t = Student’s *t*-test; * *p* < 0.05; ** *p* < 0.01; *** *p* < 0.001. Results derived from the structural equation model (SEM).

**Table 6:** Parameter estimates of final model.

Associations between Variables			RW	SE	CR	*p*
strategies_P	<-->	evaluation_P	0.133	0.006	21.919	***
strategies_O	<-->	strategies_P	0.021	0.003	6.721	***
evaluation_O	<-->	evaluation_P	0.051	0.004	12.695	***
strategies_O	<-->	evaluation_O	0.117	0.006	19.544	***
Satisfaction	<-->	strategies_P	−0.010	0.007	−1.442	0.149
strategies_O	<-->	Satisfaction	−0.007	0.007	−0.964	0.335
Satisfaction	<-->	evaluation_P	0.001	0.008	0.167	0.868
evaluation_O	<-->	Satisfaction	0.014	0.008	1.760	0.078
modalities_P	<-->	strategies_P	0.143	0.006	22.678	***
modalities_P	<-->	evaluation_P	0.100	0.005	18.213	***
strategies_O	<-->	modalities_O	0.155	0.007	23.031	***
evaluation_O	<-->	modalities_O	0.096	0.006	15.957	***
Opinion	<---	strategies_P	0.068	0.030	2.265	***
Opinion	<---	evaluation_P	−0.042	0.032	−1.330	***

Note: RW = regression weights; SE = standard error; CR = critical radio; SRW = standardised regression weights; *** *p* < 0.001.

## Data Availability

The data presented in this study are available on request from the corresponding author.
